# Historical Perspective of Pore-Forming Activity Studies of Voltage-Dependent Anion Channel (Eukaryotic or Mitochondrial Porin) Since Its Discovery in the 70th of the Last Century

**DOI:** 10.3389/fphys.2021.734226

**Published:** 2021-10-26

**Authors:** Roland Benz

**Affiliations:** Department of Life Sciences and Chemistry, Jacobs University Bremen, Bremen, Germany

**Keywords:** mitochondria, eukaryotic pore, VDAC, single channel, porin, electrophysiology, evolution

## Abstract

Eukaryotic porin, also known as Voltage-Dependent Anion Channel (VDAC), is the most frequent protein in the outer membrane of mitochondria that are responsible for cellular respiration. Mitochondria are most likely descendants of strictly aerobic Gram-negative bacteria from the α-proteobacterial lineage. In accordance with the presumed ancestor, mitochondria are surrounded by two membranes. The mitochondrial outer membrane contains besides the eukaryotic porins responsible for its major permeability properties a variety of other not fully identified channels. It encloses also the TOM apparatus together with the sorting mechanism SAM, responsible for the uptake and assembly of many mitochondrial proteins that are encoded in the nucleus and synthesized in the cytoplasm at free ribosomes. The recognition and the study of electrophysiological properties of eukaryotic porin or VDAC started in the late seventies of the last century by a study of Schein et al., who reconstituted the pore from crude extracts of *Paramecium* mitochondria into planar lipid bilayer membranes. Whereas the literature about structure and function of eukaryotic porins was comparatively rare during the first 10years after the first study, the number of publications started to explode with the first sequencing of human Porin 31HL and the recognition of the important function of eukaryotic porins in mitochondrial metabolism. Many genomes contain more than one gene coding for homologs of eukaryotic porins. More than 100 sequences of eukaryotic porins are known to date. Although the sequence identity between them is relatively low, the polypeptide length and in particular, the electrophysiological characteristics are highly preserved. This means that all eukaryotic porins studied to date are anion selective in the open state. They are voltage-dependent and switch into cation-selective substates at voltages in the physiological relevant range. A major breakthrough was also the elucidation of the 3D structure of the eukaryotic pore, which is formed by 19 β-strands similar to those of bacterial porin channels. The function of the presumed gate an α-helical stretch of 20 amino acids allowed further studies with respect to voltage dependence and function, but its exact role in channel gating is still not fully understood.

## Origin of Bacteria and Cell Organelles

The age of earth is around 4.5 billion years. Stromatolites represent presumably the oldest fossils that date back to something like 3.7 billion years (Garwood, 2012; Nutman et al., 2016). How life on earth started is still a matter of debate. One hypothesis suggests that molecules serving as the basis of life formed from atoms under the input of energy from different sources as proposed by the Miller-Urey experiment (Miller, 1953; Osinski et al., 2020; Takeuchi et al., 2020). Cell-like particles may have formed in the Hadean eon within such a primordial broth from a variety of small molecules. They represent the origin of life, which began about 4 billion years ago (Garwood, 2012; Takeuchi et al., 2020). The first organisms on earth are not known and they did not leave any evidence of their live. What we know are the oldest fossils in the form of sedimentary rocks, known as stromatolites (Margulis et al., 1986; Nutman et al., 2016). They were formed by microbial communities containing photosynthetic bacteria and cyanobacteria and represent the oldest sign for the existence of photosynthesis (Awramik, 1992; Gérard et al., 2009). It is possible that these bacteria were Gram-negative, which means that the cytoplasm was surrounded by two membranes. Their outer membranes may be considered as permeability barriers similar to those of modern Gram-negative bacteria and the mitochondrial outer membrane.

The Last Universal Common Ancestor (LUCA) is a bacteria-like organism, which existed before the bacterial cell lineage divided into the different kingdoms (Theobald, 2010; McInerney, 2016; Weiss et al., 2016). The LUCA is joint ancestor of bacteria and archaea and existed presumably within the time of begin of earth and the oldest fossils, which means that the division of the cell lineage occurred about 4 billion of years ago (Di Giulio, 2011). No fossils of this organism are known but the comparison of the genomes of all modern organisms allowed the identification of a set of about 355 genes, which could have been present in the LUCA (Weiss et al., 2016). The genes code for proteins involved in energy metabolism, synthesis of amino acids, and the equipment for transcription and translation of protein synthesis (Chioccioli et al., 2020; Koonin et al., 2020). It seems to be possible that the life of LUCA depended on hydrogen and metals favoring a deep sea vent environment for its existence (Weiss et al., 2016). Nevertheless, also other environments for the existence of LUCA are possible, for example, in ponds of different salinity and pH and in an N_2_-CO_2_ atmosphere together with dry-wet cycles and UV light (Sasselov et al., 2020). The LUCA divided subsequently into the different kingdoms of life: bacteria, eukaryote, and archaea.

## The Endosymbiotic Theory and the Development of Mitochondria From Strictly Aerobic Bacteria

A similar concept as described for the LUCA exists also for the LECA, the Last Eukaryotic Common Ancestor (Hedges, 2002; Koonin, 2015; O’Malley et al., 2019). The first eukaryotic cell represents presumably a genomic hybrid of bacteria and archaea (Katz, 2012; Grau-Bové et al., 2015). Special for the LECA is its endosymbiotic capacity because between one and 2 billion years ago specialized Gram-negative bacteria were taken up. This provided a considerable advantage for the eukaryotic host because its energy metabolism was characterized before endosymbiosis by anaerobic fermentation and is now complemented with cellular respiration (Margulis, 1981). The endosymbionts were presumably α-proteobacteria and cyanobacteria (Gray, 2012; Degli, 2014; Nowack and Weber, 2018). This can be concluded from the homology of aerobic respiration between mitochondria and α-proteobacteria and of photosynthesis between chloroplasts and cyanobacteria (Martijn et al., 2018; Nowack and Weber, 2018).

Besides the genes coding for the respiration chain, there exist also other indications for the close relation of mitochondria with Gram-negative bacteria. All proteins residing in the outer mitochondrial membrane and in the intermembrane space are encoded in the nucleus and synthesized on cytoplasmic ribosomes of the host cell and are imported post-translationally into mitochondria with modifications of amino acids in particular of cysteines (Saletti et al., 1859; Grevel et al., 2019). This suggests that since the event of endosymbiosis about 1–2 billion years ago many genes of the protomitochondrion came under control of the eukaryotic host. The mitochondrial proteins produced in the cytoplasm of the host cells are imported *via* the mitochondrial outer membrane import TOM complex into the intermembrane space using a not yet fully identified import signal, which is presumably related to the β-strands of the imported proteins (Bausewein et al., 2020; Drwesh and Rapaport, 2020; Moitra and Rapaport, 2021). This is based on the observation that the β-hairpin element of eukaryotic porins interacts with the mitochondrial import receptor Tom20 (Jores et al., 2016). From there, the mitochondrial outer membrane proteins are assembled by the multi-subunit TOB/SAM system (sorting and assembly machinery) in the mitochondrial outer membrane with the help of the hexamer of the small TIM chaperones (Moitra and Rapaport, 2021). The SAM system has a high homology to the barrel-assembly machinery BAM of Gram-negative bacteria, which provides also evidence that Gram-negative bacteria were ancestors of mitochondria (Pereira and Lupas, 2018; Diederichs et al., 2020, 2021; Takeda et al., 2021). Accordingly, the bacterial outer membrane represents the ancestor of the mitochondrial outer membrane. However, whereas the porins of the bacterial outer membranes have only passive sieving properties in bacterial metabolism, it seems that the mitochondrial outer membrane including the eukaryotic porin or Voltage-Dependent Anion Channel (VDAC) plays also an active and important role in mitochondrial and cellular metabolism (Benz, 1994a,b; Shoshan-Barmatz et al., 2006, 2010; Gatliff and Campanella, 2012; Grevel and Becker, 2020). It binds different kinases (Fiek et al., 1982; Brdiczka, 1990; Adams et al., 1991; De Pinto et al., 2003) and evidence has been provided that eukaryotic porins play also an important role in mitochondria-mediated apoptosis, protein translocation, and are also involved in response to drugs (Shoshan-Barmatz et al., 2010; Grevel et al., 2019; Grevel and Becker, 2020). This applies also to its interaction with the 18kDa translocator protein, also known as peripheral benzodiazepine receptor, which mediates cholesterol transport between mitochondrial membranes, cytochrome C release, and apoptosis (Gatliff and Campanella, 2012; Bader et al., 2019; Betlazar et al., 2020).

## A Voltage-Dependent Pore was Discovered in Crude Extracts of Paramecium Mitochondria

The most interesting property of eukaryotic porins or VDACs is their voltage dependence, which is clearly no reconstitution artifact. The voltage dependence of eukaryotic porins was first discovered in extracts of *Paramecium aurelia* mitochondria by Schein et al. (1976). They dissolved mitochondrial-rich fractions from *Paramecium* supplemented with asolectin in hexane and used this lipid-protein mixture for membrane formation. This means that they spread the crude protein-lipid mixture on the surface of electrolyte solutions in a Teflon cell. Membranes were then formed from the protein-lipid layers across a small hole in a thin Teflon by the Montal-Mueller method (Montal and Mueller, 1972). Schein et al. (1976) observed frequently high conductance channels/pores in these solvent-depleted membranes. The number of reconstituted channels in the folded membranes was somewhat dependent how much from the mitochondria-rich fractions were present in the lipid-protein mixtures (Schein et al., 1976). Highest pore-forming activity was observed in fractions 50–55 of the sucrose gradient. The open channels had a conductance of 200 pS in an asymmetric solution of 20mM MgCl_2_ versus 20mM CaCl_2_, or 750 pS in an asymmetric solution of 1M KCl versus 0.1M KCl (Schein et al., 1976). The current-voltage behavior of the channel was ohmic, resulting in a linear current-voltage curve (Schein et al., 1976).

**Figure 1:**
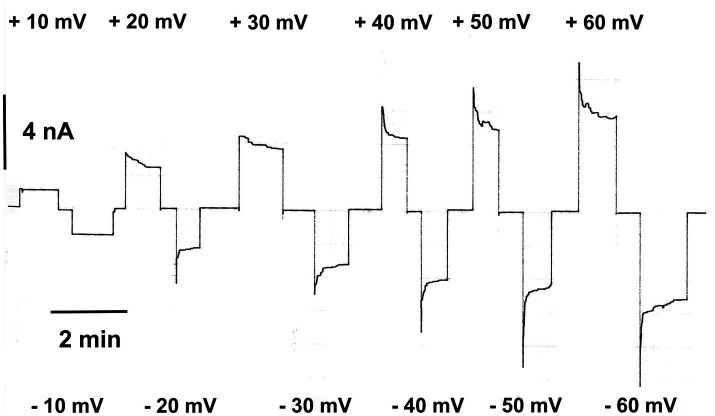
Relaxation of the membrane current in the presence of eukaryotic porin 1 from humans (hVDAC1, Porin 31HL; Benz et al., 1992). The membrane potential was first switched to +10mV and then to −10mV applied to the cis-side of the membrane containing about 50 hVDAC1 pores. Note that the membrane current did not decrease at these voltages. Then, higher positive and negative voltages were applied which resulted in a substantial exponential decrease of the membrane current. The membrane was formed of diphytanoyl phosphatidylcholine/n-decane. The aqueous phase contained 0.5M KCl (pH 7.2); T=20°C.

At voltages smaller than 10mV, the channels were frequently in the “open” configuration. However, they switched a higher voltages in closed configurations, which could be observed in experiments when the voltage was switched to ±20mV. The maximum voltage dependence was reached at about ±40 to ±50mV transmembrane potential and appeared to be symmetric with respect V_m_=0mV (Schein et al., 1976). Figure 1 shows an example of the voltage dependence of human eukaryotic porin 1 (hVDAC1) reconstituted into solvent-containing membranes made of diphytanoyl phosphatidylcholine/n-decane membranes. The experiment started by an application of +10mV to about 50 reconstituted hVDAC1 pores, followed by application of −10mV. At ±20mV applied to the membrane, the current through the pores started to decrease, higher positive, and negative voltages resulted in a stronger decrease of the current and a faster exponential decay from the initial current to the final current level at longer times (see Figure 1). The closing of the eukaryotic porin is a relatively slow process as Figure 1 clearly demonstrates. The invers process, i.e., the reopening of the pores, when the voltage is switched off is much faster, which means that it is difficult to measure it precisely (Schein et al., 1976). It is sometimes so fast that it cannot be resolved properly (Colombini, 1979; Benz, 2004).

The decrease of the current at higher positive and negative voltages than ±10mV could be analyzed using a similar approach as proposed by Schein et al. (1976) assuming a Boltzmann distribution of the open and closed states of the pore:

$$N_{o}/N_{c}=\exp\left(\frac{nF\left(V_{m}-V_{0}\right)}{RT}\right)$$ (1)

Where F, R, and T are Faraday’s constant, gas constant, and absolute temperature, respectively; n is the number of gating charges moving through the entire membrane potential. *V_0_* is the midpoint potential, where one half of the pores are open and the other half are closed, i.e., $N_{o}/N_{c}$ = 1. The open to closed ratio of the pores is given by the analysis of the experimental results of experiments similar to Figure 1 according to:

$$N_{o}/N_{c}=\left(G-G_{\min}\right)/\left(G_{0}-G\right)$$ (2)

*G* is the membrane conductance at a given membrane potential *V_m_*. *G_0_* and *G_min_* are the conductance at zero voltage, when all pores are in the open configuration and when all pores are in the closed one at very high voltage, respectively. The use of the Boltzmann distribution allows also the derivation of the activation energy for the voltage-dependent gating process of eukaryotic porins (Schein et al., 1976). The activation energy is given by *W(V_m_)*, which is equivalent to the energy of one mole pores between the open and the closed configuration, i.e., it has the form (Schein et al., 1976):

$$N_{o}/N_{c}=\exp\left(-\frac{W\left(V_{m}\;\right)}{RT}\right)$$ (3)

Comparison of Eqs. (1) and (3) shows that *W(V_m_)* is given by *nF(V_m_-V_0_)*, which means that the activation energy *nFV_0_* is about 5.8kJ/mol (1.38kcal/mol), which is a very low energy that is needed to shift the eukaryotic pores from the open into the closed configuration.

**Figure 2:**
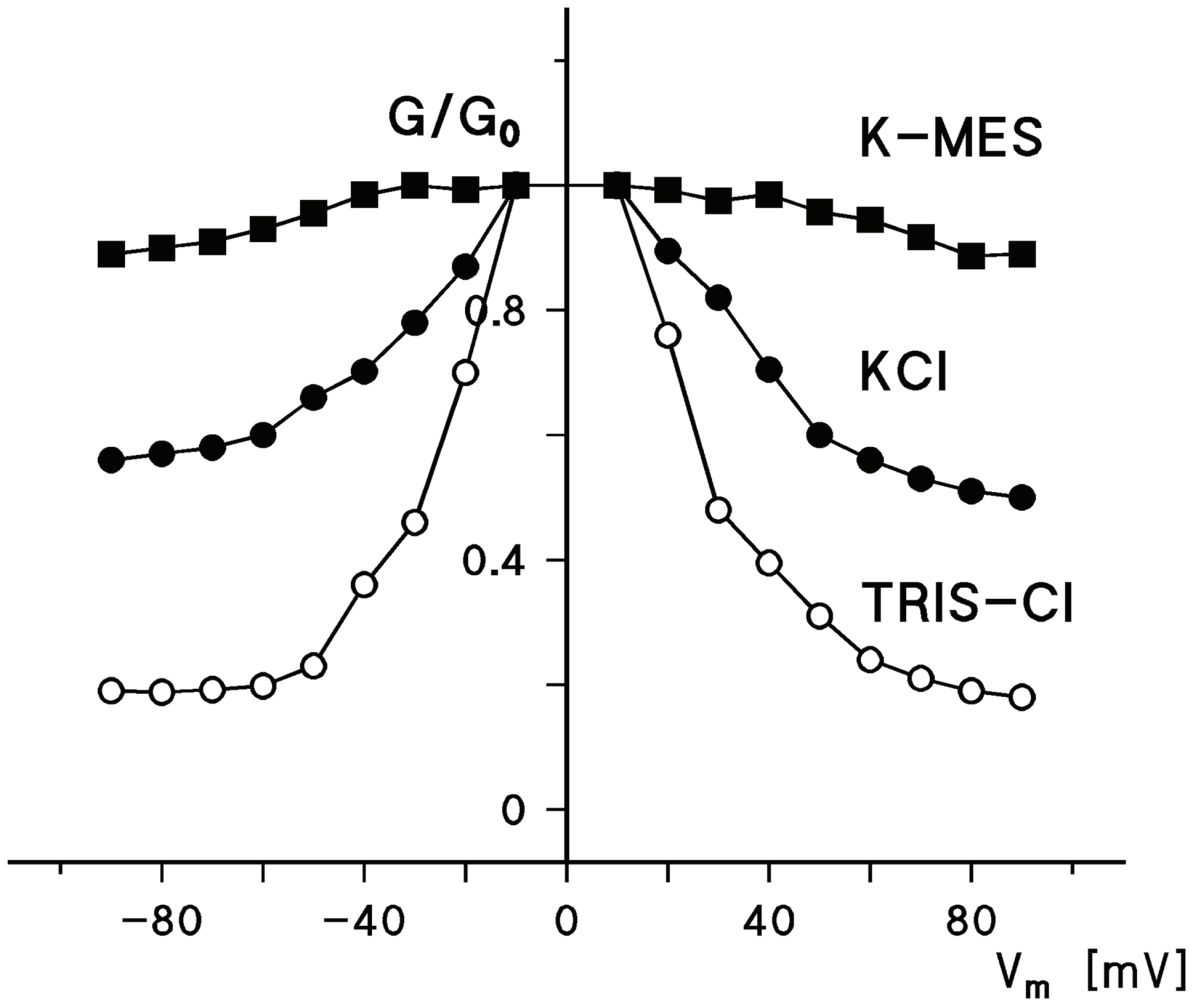
Ratio of the conductance, G, at a given voltage, V_m_, divided by the conductance, G_0_, at 10mV as a function of the voltage. The aqueous phase contained either 0.5M KCI, 0.5MK-MES, or 0.5M TRIS-HCI (pH in all cases 7.2). The cis-side contained about 10ng/ml hVDAC1 [Porin 31 HL (Benz et al., 1992)]. The sign of the voltage is given with respect to the cis-side, the side of the addition of Porin 31HL.

Figure 2 shows the ratio of the conductance, *G*, at voltages between ±10 and±90mV divided by the initial conductance *G_0_* as a function of the applied voltage for experiments similar to that shown in Figure 1 for 0.5M KCl, 0.5MK-MES, and 0.5M TRIS-Cl (mean values of three experiments taken under the same conditions). The combination of the cations and anions was chosen to show the voltage dependence of human eukaryotic porin 1 (hVDAC1, Porin 31HL) in dependence of cations and anions of different mobility because this provides not only some information on voltage dependence but also on ion selectivity of the open and closed states of the pore. It is obvious that the voltage dependence was in all cases similar. However, *G/G_0_* was found to be dependent on the type of the cation and anion present in the aqueous solution, indicating a selectivity change when the pores switched in the closed configuration at higher voltages. The exact value for the permeability ratio of potassium and chloride was difficult to obtain because the mobility of TRIS and MES inside the pore is not known. However, because of the comparably small mobility of TRIS and MES in the aqueous phase, it is possible that P_cation_/P_anion_ of the closed hVDAC1 pore is very high (around 10) in contrast to the selectivity of the open pore where P_cation_/P_anion_ is about 0.5 (Benz et al., 1992). The selectivity of the closed state may be even higher if it is impermeable for anions. On the other hand, it is also evident from Figure 2 that potassium is almost equally mobile through the open and the closed state, because of the low mobility of MES in the aqueous phase. This represents another proof that the closed state has completely different properties for the permeation of charged solutes than the open state.

**Figure 3:**
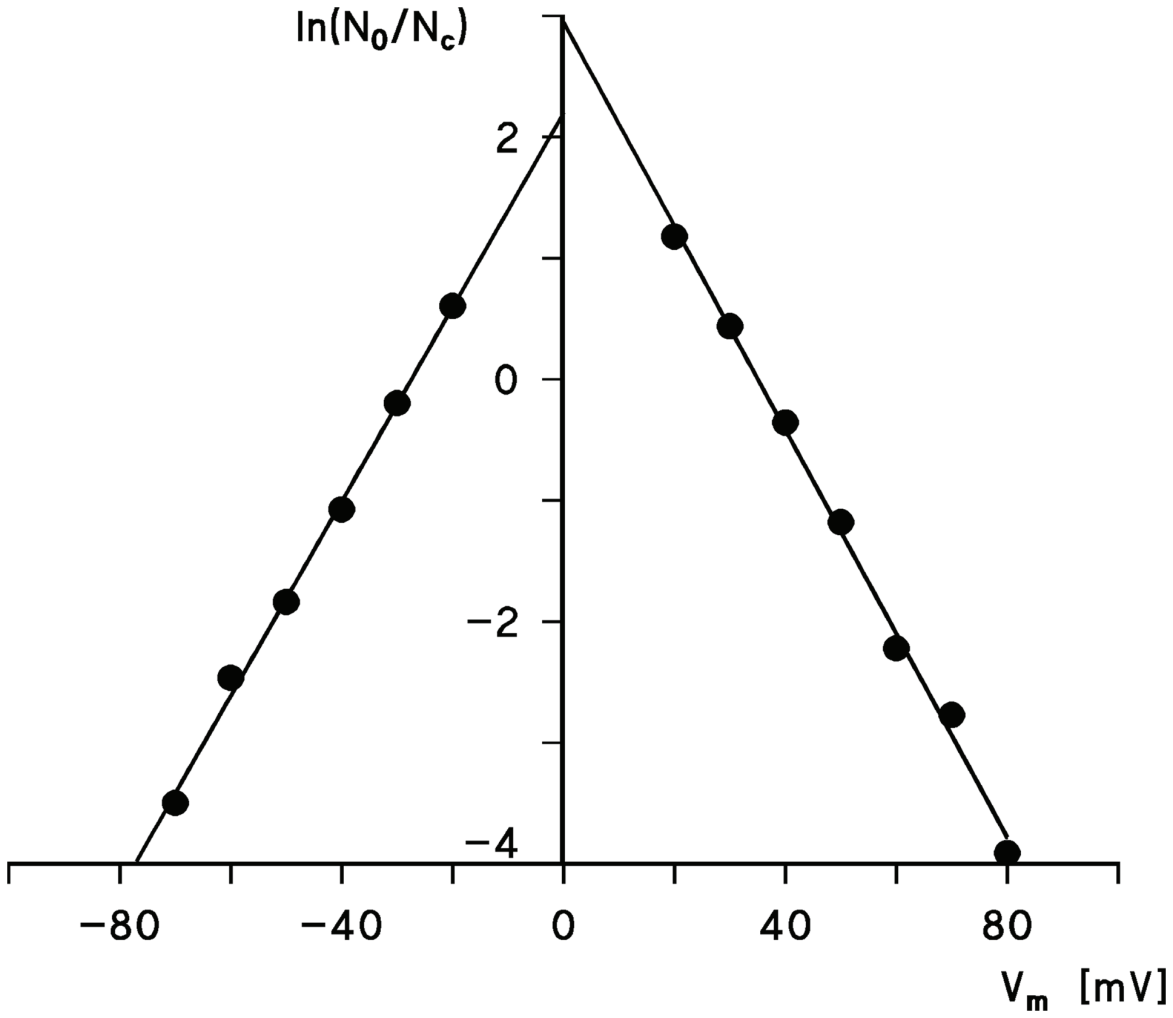
Semilogarithmic plot of the ratio, *N_0_/N_c_*, as a function of the transmembrane potential V_m_. The data were taken from Figure 2. The slope of the straight lines is such that an e-fold change of *N_0_/N_c_* is produced by a change in V_m_ of 12.5mV (left side) and 11.9mV (right side), corresponding to gating charges, n=2.0 and 2.1, respectively. The midpoint potential of the *N_0_/N_c_* distribution (i.e., N_0_=N_c_) was at 27.4mV (left side) and 35mV (right side).

The voltage dependence of the pores formed by Porin 31HL (hVDAC1) can be analyzed using Eq. (1) and semilogarithmic plots of the ratio *N_0_/N_c_* as a function of the transmembrane potential, V_m_, calculated from the experimental results according to Eq. (2) as shown in Figure 3. The slope of the straight line for the application of negative voltages for an e-fold change in *N_0_/N_c_* was about 12.5mV, which suggested that the number of charges involved in the gating process was approximately 2.0. A similar analysis for positive voltages resulted in a slope for an e-fold change of *N_0_/N_c_* of 11.9mV, which suggested that the gating charge of the right branch of the voltage dependence of hVDAC1 is about 2.1. The midpoint potentials of the two *N_0_/N_c_* distributions for negative and positive voltages with respect to the addition of porin 31HL (human eukaryotic porin 1) were−27.4mV and+35mV, respectively. This indicated a slight asymmetry in the midpoint potential, V_0_, where the number of open and closed channels was balanced, i.e., *N_0_/N_c_*=1. It is noteworthy that the voltage dependence of eukaryotic porin (VDAC) in the first study of *Paramecium* mitochondria exhibited a much higher voltage dependence because n was about 4.5 and the midpoint potentials of *N_0_/N_c_* were around ±20mV (Schein et al., 1976). In a more recent study of eukaryotic porin of *Paramecium tetraulia*, where the eukaryotic porin was purified to homogeneity, the voltage dependence of the reconstituted pore was lower with about 2 gating charges and a midpoint potential for *N_0_/N_c_* of 32mV (Ludwig et al., 1989).

The voltage dependence of eukaryotic porins from a variety of eukaryotic organisms was investigated in detail in many studies: *Paramecium* (Schein et al., 1976; Doring and Colombini, 1984; Ludwig et al., 1989); Mammals: rat (Roos et al., 1982; Colombini, 1983; Ludwig et al., 1986), rabbit (De Pinto et al., 1987a), bovine (De Pinto et al., 1987a), pig (De Pinto et al., 1987a), and human brain (Bureau et al., 1992); Fish: *Anguilla anguilla* (De Pinto et al., 1991c); Plants: potato (Heins et al., 1994; Lopes-Rodrigues et al., 2020), pea (Fischer et al., 1994), corn (Smack and Colombini, 1985; Aljamal et al., 1993; Fischer et al., 1994), wheat (Blumenthal et al., 1993), and pea root plastid porin (Fischer et al., 1994; Popp et al., 1997); Other organisms: *Neurospora crassa* (Freitag et al., 1982c), yeast (Ludwig et al., 1988), and *Dictyostelium* (Troll et al., 1992); and Flies: *Protophormia* (Wiesner et al., 1996) and *Drosophila* (De Pinto et al., 1989a; Aiello et al., 2004; Komarov et al., 2004). Common to all of these studies is that the eukaryotic porins of all these eukaryotes formed high-conducting channels in reconstituted systems. They were all in their open configuration at small transmembrane voltages smaller or equal to 10mV (Benz, 1994b). At higher voltages, they switched into substates. The analysis of the voltage dependence in terms of the above used formalism showed that the number of gating charges for almost all pores formed by these eukaryotic porins was around two, which means that an e-fold change in *N_0_/N_c_* occurred, when the voltage across the membrane was changed by about 12 mV (De Pinto et al., 1987a; Benz, 1994b, 2004). The midpoint potential for the distribution of the open and closed pores (i.e., *N_0_ =N_c_*) was in many cases either symmetrical or slightly asymmetrical with values around ±30mV to ±40mV (De Pinto et al., 1987a; Benz, 1994b, 2004).

## Isolation and Purification of Eukaryotic Porins

Schein et al. (1976) had already the idea that voltage-dependent pore was present in the mitochondrial outer membrane of the *Paramecium* mitochondria, because of its high permeability. This was revealed in a study by Colombini (1979), where he could explicitly show that the pore-forming activity came from the outer membrane of rat liver mitochondria, but not from fractions containing inner membranes. The pore had a conductance of 0.45 nS and 4.5 nS in 0.1 and 1M KCl, respectively. The first biochemical evidence for the identity of the pores in the mitochondrial outer membranes of rat liver and mung beans was provided by Hiroshi Nikaido and coworkers (Zalman et al., 1980). In analogy to their work with bacterial porins, they were able to reconstitute fragments of the mitochondrial outer membranes into vesicles from soybean lipids and demonstrated that the vesicles became permeable for low molecular mass carbohydrates but not for high molecular mass dextrans. Following different biochemical procedures, Zalman et al. (1980) were able to identify a protein in the mitochondrial outer membranes of mung bean mitochondria with a molecular mass of about 30kDa, which obviously was responsible for the permeability properties of the reconstituted vesicles. It is quite difficult to establish a potential across vesicles membranes or to study the permeability of charged solutes in the liposome system, which means that the putative voltage dependence of the pore could not be studied. Nevertheless, the diffusion of carbohydrates with a molecular mass up to 8kDa suggested indeed that Zalman et al. (1980) identified the eukaryotic porin of mung beans as a general diffusion pore.

A similar study was performed by Mihara et al. (1982) to identify yeast porin. They isolated yeast porin as a 29kDa polypeptide from the mitochondrial outer membranes of yeast mitochondria. To verify their results, they demonstrated that yeast porin was not accessible for protease treatment as long the protein was localized in the mitochondrial outer membrane (Mihara et al., 1982). When the protease digestion was performed in the presence of detergent yeast porin was no longer protected. They were also the first to notice that *in vitro*-synthesized yeast porin using yeast total RNA had the same molecular mass as the native protein and did not exhibit any additional leader sequence. It was incorporated directly into intact mitochondria and not into rough endoplasmic reticulum (Mihara et al., 1982). A membrane potential across the inner mitochondrial membrane was not important for this process (Mihara et al., 1982). A similar conclusion was obtained from the import of porin from *N. crassa* synthesized in homologous or heterologous cell-free systems into mitochondria (Freitag et al., 1982b).

The identification of other eukaryotic porins also proceeded at the same time. Roos et al. (1982) were the first to identify a mammalian eukaryotic porin from rat liver. Rat liver mitochondria were sub fractionated. When the OM fraction obtained by centrifugation steps was treated with detergent it showed pore-forming activity in artificial lipid bilayer membranes (Roos et al., 1982). Rat liver porin was identified as a 32kDa protein using different biochemical methods and the reconstitution of the protein into artificial lipid bilayers. Rat liver porin formed voltage-dependent pores in lipid bilayers with a single-channel conductance of about 4 nS in 1M KCl, which is typical for eukaryotic porins (Colombini, 1979, 1980, 1983). The molecular mass of rat liver porin was confirmed by other groups (Lindén et al., 1982a; Colombini, 1983). However, in contrast to a putative purification of rat liver porin by a Concanavalin A-containing column, eukaryotic porins are pure polypeptides (Benz, 1994b), which means that the 300-fold purification of rat liver porin (rVDAC) by chromatography across this column has presumably nothing to do with eukaryotic porins as glycoproteins Colombini, 1980, 1983).

The purification of eukaryotic porins until 1982 was always dependent on the fractionation of the mitochondrial membranes using different methods, such swelling and shrinking of mitochondria followed by density gradient centrifugation (Roos et al., 1982; Lindén et al., 1982a). This procedure and related methods were always accompanied by a substantial loss of outer membrane material because it is in part tightly associated with the mitochondrial inner membrane (van der Laan et al., 2016). This means that it was a considerable breakthrough when eukaryotic porins could be isolated from detergent-solubilized whole mitochondrial membranes of *N. crassa* by using the method of Freitag et al. (1982a). In a first step, mitochondria were lysed by an osmotic shock and the total mitochondrial membranes were obtained by centrifugation. Next, the detergent-solubilized mitochondrial membrane proteins were applied to a dry hydroxyapatite (HTP) column and the eluate was passed in a second step through a dry HTP/celite column in a ratio of 1:1 (w/w). Using this method, *N. crassa* porin was almost pure (Freitag et al., 1982a). Following the isolation and purification of different eukaryotic porins, the method was refined (De Pinto et al., 1987b). Finally, the mitochondrial membrane proteins were dissolved in 3% Triton X-100 using a low protein/detergent ratio and then passed only once through a dry HTP/celite column in a ratio of 2:1 (w/w) (De Pinto et al., 1987b). This procedure resulted in eukaryotic porins in particular from mammals of high purity and was successfully applied many times to the purification of eukaryotic porins by the Bari/Catania group of research into mitochondria (Ludwig et al., 1986, 1988; De Pinto et al., 1987a,b; Carbonara et al., 1996). This easy purification method allowed also further investigations of structure and function of eukaryotic porins and their interaction with different detergents (De Pinto et al., 1989b, 1991a,b; De Pinto and Palmieri, 1992).

## Primary Sequences of Eukaryotic Porins

The first two primary sequences of eukaryotic porins that became known were those of yeast and *N. crassa* (Mihara and Sato, 1985; Kleene et al., 1987). Mammalian porins could not be sequenced from their cDNA at that time because their sequence is only distantly related to the porins of the microorganisms despite a similar length and a relative large fraction of hydrophilic amino acids. That was possible, when human porin (hVDAC1, porin 31HL) was sequenced by direct amino acid sequencing (Kayser et al., 1989). Shortly after, eukaryotic porins from higher eukaryotic cells could be cloned in different organisms, such as mouse and humans (Blachly-Dyson et al., 1993, 1994; Ha et al., 1993; Sampson et al., 1996). Mammalian genomes contain the genes coding for three VDAC species. The genes have the same exon-intron structure (Young et al., 2007). The proteins have the same length but they exhibit some differences, in particular in the number of cysteines. The differences in the primary sequence of the three VDAC isoforms in mammals did not alter the structure of the splicing sequences and the organization of the three genes (Pinto and Messina, 2004; Young et al., 2007; De Pinto, 2021). The most abandoned version of the VDAC isoforms is VDAC1, which was extensively studied *in vivo* and *in vitro* (Benz, 1994b, 2004; Báthori et al., 2006; Shoshan-Barmatz et al., 2020). However, also the other two human isoforms were studied in recent years (Checchetto et al., 2014; Gattin et al., 2015; Queralt-Martín et al., 2020). The results of these studies were to some extent controversial, because hVDAC3 was a small channel-forming component in one of the studies (Checchetto et al., 2014), whereas it has in a more recent study quite normal electrophysiological properties (Queralt-Martín et al., 2020). This means that the three human eukaryotic porins have a similar single-channel conductance (see Table 1) and the pores formed by the three human isoforms are all voltage-dependent with some modifications (Benz, 2004; Gattin et al., 2015; Queralt-Martín et al., 2020). The expression of the isoforms may be tissue-specific but their function in mitochondrial outer membrane permeability and in other cellular important functions, such as apoptosis, is still a matter of debate (De Pinto et al., 2016; Shoshan-Barmatz et al., 2020; Shimizu et al., 2021). Three genes coding for analogs of VDAC1 have not only be found in mammals but also in the genome of the fruit fly *Drosophila melanogaster* (Aiello et al., 2004; Komarov et al., 2004). All of them with one exception code for pore-forming proteins with properties similar to most eukaryotic porins with some modification of the voltage dependence (Aiello et al., 2004; Komarov et al., 2004). Careful analysis of the genes and their comparison with those of other eukaryotic porins from insects suggested that the genes evolved by duplication from an ancestral gene (Komarov et al., 2004).

The genetic organization of eukaryotic porins in plants appears to be even more complicated (Kusano et al., 2009; Homblé et al., 2012). The genome of the popular model organism in plant biology and genetics, *Arabidopsis thaliana,* contains at least four or five genes coding for eukaryotic porin-like proteins (Lee et al., 2009; Tateda et al., 2011; Berrier et al., 2015). A similar number of eukaryotic porin genes has been found in *Lotus japonicus* and soybean, where also up to five genes were found (Wandrey et al., 2004). Localization analysis of the different isoforms of eukaryotic porins in *Arabidopsis* indicated specific functions of the porins including DNA and RNA transport (Tarasenko et al., 2021). Some of the plant porins have similar subcellular localizations (Tateda et al., 2011, 2012). Knockout mutants of AtVDAC2 and AtVDAC4 show despite similar subcellular localizations severe defects in growth indicating that these eukaryotic porins have an important function in *Arabidopsis* (Tateda et al., 2012). Many eukaryotic porins from plants have been studied in lipid bilayer membranes (see Table 1). Their single-channel conductance seems to be a little smaller than those of porins from other organisms, but plant porins show similar voltage dependences as most eukaryotic porins with some modifications. From the many porins of *Arabidopsis,* only AtVDAC3 has been studied in lipid bilayers in some detail (Berrier et al., 2015). Again, its properties were quite similar as found for most eukaryotic porins,

**Table 1:** Single-channel conductance of eukaryotic (mitochondrial) porins (VDACs) from different eukaryotic organisms in 1M KCI, pH 6, if not indicated otherwise.

Eukaryotic porin (VDAC)	G (nS)	References
Human VDAC1 (Porin 31HL)	4.3 4.1	Benz et al., 1992 Blachly-Dyson et al., 1993
Human VDAC2	4.0 2.0 and 4.0	Blachly-Dyson et al., 1993 Gattin et al., 2015
Human VDAC3	3.9	Queralt-Martín et al., 2020
Rat liver	4.3	Roos et al., 1982
Beef heart	4.0	Benz et al., 1985
Rabbit liver	4.0	Benz et al., 1985
Rat brain	4.0	De Pinto et al., 1987a
Rat kidney	4.0	De Pinto et al., 1987a
Pig heart	3.5	De Pinto et al., 1987a
*Anguilla anguilla*	4.0	De Pinto et al., 1991c
*Drosophila melanogaster* VDAC CG17140	4.5 3.4/1M NaCl	De Pinto et al., 1989a Komarov et al., 2004
*Protophormia*	4.5	Wiesner et al., 1996
*Neurospora crassa*	4.5	Freitag et al., 1982c
Yeast	4.5 4.2 4.2	Forte et al., 1987a Ludwig et al., 1988 Blachly-Dyson et al., 1993
*Paramecium*	4.5 2.4	Colombini, 1979 Ludwig et al., 1989
*Pea* mitochondria	1.5 and 3.7	Schmid et al., 1992
*Pea* root plastids	1.5 and 3.7	Fischer et al., 1994
*Maize* root plastids	1.5 and 3.7	Fischer et al., 1994
*Solanum tuberosum* POM 34	2.0 and 3.5	Heins et al., 1994
*Maize* mitochondria	1.5 and 3.7	Carbonara et al., 1996
*Phaseolus coccineus*	3.7	Krammer et al., 2014
*Arabidopsis*	0.5/300mM KCl	Berrier et al., 2015

*The pores were measured at low transmembrane potentials where almost all pores should be in their open configuration. All pores formed by these proteins were anion selective in their open state* (i.e.*, at small transmembrane voltage). If not indicated otherwise, the single-channel conductance of the eukaryotic porins refers to VDAC1.*

## Identification of Eukaryotic Porin as Dicyclohexylcarbodiimide (Dccd)-Binding Protein in the Mitochondrial Outer Membrane

When pig heart mitochondria are treated with low doses (1.5nmol/mg of mitochondrial protein) of C14-labeled dicyclohexylcarbodiimide (DCCD), three mitochondrial polypeptides of approximately 9, 16, and 33kDa bound DCCD (Houstĕk et al., 1981; De Pinto et al., 1985). The two smaller DCCD-binding proteins are parts of the F_0_F_1_ ATPase localized in the mitochondrial inner membrane (Houstĕk et al., 1981). The 33kDa DCCD-binding protein present in the mitochondrial outer membrane of pig heart was identified as the eukaryotic porin based on biochemical evidence and electrophysiological experiments although DCCD-binding did not change the characteristics of the pore (De Pinto et al., 1985). However, labeling of porin with DCCD resulted in the loss of hexokinase binding to porin (Nakashima et al., 1986; Nakashima, 1989), because porin is the hexokinase-binding protein (Fiek et al., 1982; Lindén et al., 1982b). Fifty percent inhibition of hexokinase binding occurred at very low levels of DCCD by less than 2nmol of DCCD/mg of mitochondrial protein (Nakashima, 1989). Water-soluble carbodiimides had no effect on hexokinase binding on porin, indicating that the binding place was in a hydrophobic environment. DCCD-binding to proteins suggested that a negatively charged amino acids exist in a hydrophobic environment (De Pinto et al., 1985; Nakashima, 1989). This amino acid was identified as glutamate 72 in the sequence of bovine heart eukaryotic porin (De Pinto et al., 1993). The role of this negative charge in mitochondrial metabolism of the three VDAC isoforms in Zebrafish was studied in detail recently because the homologous glutamate 73 is present in VDAC1 and VDAC2 but not in VDAC3 and plays an important role in regulation of Ca2+ uptake (Shimizu et al., 2021). Diafenthiuron is an acaricide and insecticide developed by Ciba-Geigy that cannot longer be used as a pesticide because of its toxicity (Kayser and Ellinger, 2001). Its active form is the carbodiimide CGA 140'408, which labels also components of the mitochondrial ATPase together with the eukaryotic porin of the fly *Protophormia* (Wiesner et al., 1996). Reconstitution experiments with the CGA 140'408-modified porin of *Protophormia* showed also no significant effects on the characteristics of channel formation by *Protophormia* porin similar as described above for the binding of DCCD to pig heart porin (De Pinto et al., 1985; Wiesner et al., 1996).

## Reconstitution of Eukaryotic Porins in Lipid Bilayer Membranes

The first reconstitution of eukaryotic porin (VDAC) occurred *via* the enrichment of mitochondrial particles from *Paramecium* mitochondria with endogenous asolectin followed by the formation of solvent-depleted membranes according to the Montal-Mueller method (Montal and Mueller, 1972). For this, the protein-lipid mixtures were spread with hexane on the aqueous surface of the membrane cell for membrane formation (Schein et al., 1976). The pores were present in the membranes immediately after its formation. The number of pores incorporated into the membranes by this method depended on the concentration of porin in the protein-lipid mixtures (Schein et al., 1976). The reconstitution of the eukaryotic porins occurred late on similarly as the incorporation of bacterial porins into lipid bilayers (Benz et al., 1978). After isolation and purification of eukaryotic porins, they were added to the aqueous electrolyte solution bathing preexisting membranes (Colombini, 1979; Roos et al., 1982; Freitag et al., 1982c) or to preexisting lipid vesicles (Zalman et al., 1980; Mihara et al., 1982). The characteristics of the pores formed by eukaryotic porins were not dependent on the method of bilayer formation, either painted of folded, although this was occasionally claimed (Colombini, 1983). It seems that these pores, similar as bacterial porins form their own sphere and their properties, are only little dependent on the lipid environment in the membranes.

**Figure 4:**
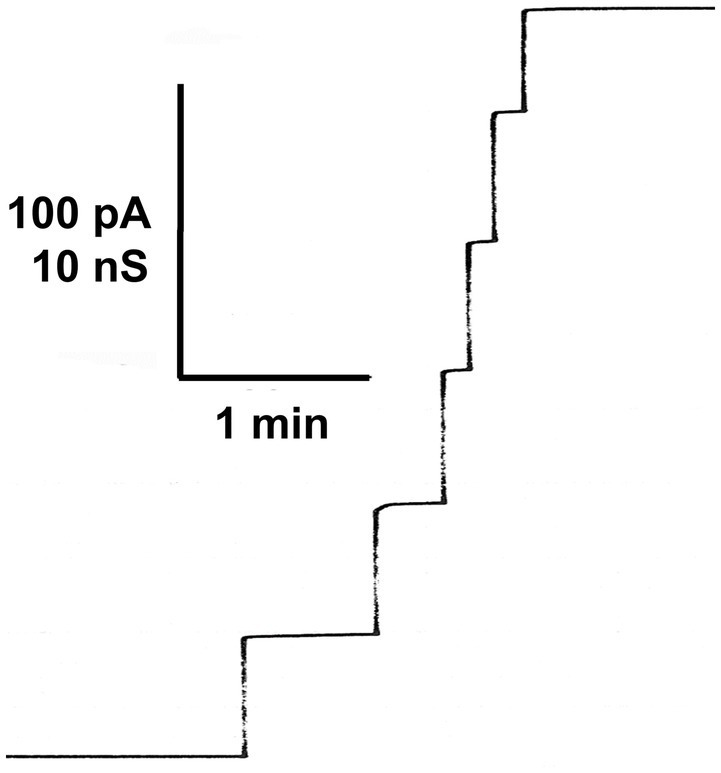
Stepwise increase of the membrane current (given in pA) after the addition of porin 31HL (hVDAC1) to a black lipid bilayer membrane given as a function of time. The aqueous phase contained 5ng/ml Porin 31HL and 1M KCl (Benz et al., 1992). The membrane was formed from diphytanoyl phosphatidylcholine/n-decane. The voltage applied was 10mV; T=20°C.

Figure 4 shows the stepwise increase of membrane current when a eukaryotic porin (Porin 31HL) was added to the aqueous 1M KCl solution bathing a painted black lipid membrane from diphytanoyl phosphatidylcholine/n-decane. The steps were mostly directed upward at low transmembrane potential, as it was already outlined above, when the voltage dependence of the same eukaryotic pore was discussed. The single-channel conductance of Porin 31HL was under these conditions about 4 nS (see the histogram of the current fluctuations obtained with Porin 31HL).

**Figure 5:**
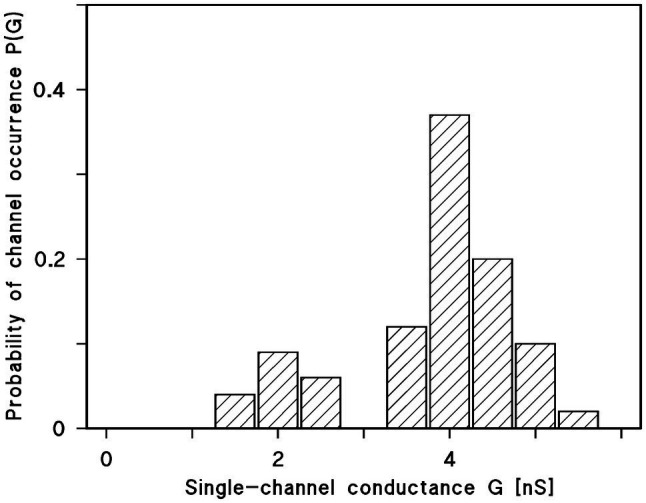
Histogram of conductance fluctuations observed with membranes of diphytanoyl phosphatidylcholine/n-decane in the presence of Porin 31HL (Benz et al., 1992). P(G) is the probability for the occurrence of a conductance step with a certain single-channel conductance (given in nS). The aqueous phase contained 1M KCl. The voltage applied was 10mV. The mean value of all upward directed steps was 4.3 nS for the right-side maximum and 2.4 nS for the left-side maximum (in total 288 single events); T=20°C.

The histogram of all current fluctuations observed with Porin 31HL showed two maxima for pore distribution (Benz et al., 1992). One is centered on 4 nS and the other one around 2 nS (Figure 5). The higher single-channel conductance refers presumably to the first opening of a Porin 31HL pore by reconstitution of one pore protein into the membrane. The lower conductance represents pores that adopted a sublevel of conductance and reopened again, i.e., these pores reflect the closed state of the Porin 31HL pore, which seems to be around 2 nS.

Similar experiments were performed with many eukaryotic porins in different research groups. Typical for the pores formed by the eukaryotic porins is an open state between 4 and 4.5 nS at small voltages. However, histograms of all current fluctuation show also pores around 2 nS, which could refer to substates of the open pore. Plant porins have a somewhat smaller conductance just a little below 4 nS at low voltage. In the case of plant porins, also a second maximum was observed at 1.5 nS. The hypothesis is that this maximum also referred to the closed state or voltage-gated substates of the mitochondrial pore.

## Ionic Selectivity of the Open State of Pores Formed by Eukaryotic Porins

The single-channel conductance of pores formed by eukaryotic porins followed in salts composed of different cations and anions approximately the mobility of the different ions in the aqueous phase (Colombini, 1979; Roos et al., 1982). This result suggested together with the high single-channel conductance (see Table 1) that eukaryotic porin pores are wide and water filled. Nevertheless, the pores showed some preference for anions. The ionic selectivity of channels/pores reconstituted into artificial lipid bilayers can be measured by the zero-current membrane potential as the result of a salt gradient applied across the membranes. This could be performed by using a high impedance electrometer connected to electrodes with salt bridges when the gradient is established across the membranes (Benz et al., 1979). It is also possible to measure current-voltage curves and extrapolating the current to zero. The corresponding voltage provides the same information (Schein et al., 1976). Table 2 shows examples of zero-current membrane potentials obtained for several eukaryotic porins using salts composed of anions and cations of different mobility in the aqueous phase. It is evident from the data in Table 2 that the mobility of the ions in the aqueous phase has a substantial influence on the zero-current membrane potential of the pores and the permeability ratio P_anion_/P_cation_. For KCl that is composed of equally mobile potassium ions and chloride, the pores are slightly anion selective. This changes remarkably, when potassium ions or chloride are replaced by lithium ions and acetate, respectively. For LiCl, the potential is more negative, whereas it gets positive for K-acetate. This result is typical for general diffusion pores similar to general diffusion pores of the bacterial outer membranes (Benz, 1994a). The voltage dependence of the eukaryotic porins changes this picture, because all eukaryotic porins are cation-selective in the closed state (Benz, 1994b, 2004). The switch from open-anion selectivity to closed-cation selectivity of eukaryotic porins adopts presumably an important role in regulation of mitochondrial energy metabolism.

**Table 2:** Zero-current membrane potentials, V_m_, of lipid bilayer membranes the presence of rat liver, yeast, human eukaryotic porin1, human eukaryotic porin2, and *Paramecium* porins measured for 10-fold gradients of different salts. V_m_ is defined as the potential of the dilute side (10mM/100mM) relative to that of the concentrated side (100mM/1M); P_anion_/P_cation_ was calculated from the Goldman-Hodgkin-Katz equation (Benz et al., 1979).

Salt	V_m_ [mV] 10-fold gradient	P_anion_/P_cation_	References
Rat liver			
KCl (pH 6)	−11	1.7	Roos et al., 1982
LiCl (pH 6)	−24	3.4	Roos et al., 1982
Potassium acetate(pH 7)	+14	0.50	Roos et al., 1982
Yeast			
KCl (pH 6)	- 7	1.4	Ludwig et al., 1988
KCL	- 11	1.8	Forte et al., 1987a
KCl	- 11	1.8	Blachly-Dyson et al., 1993
LiCl (pH 6)	−20	2.6	Ludwig et al., 1988
Potassium acetate (pH 7)	+14	0.5	Ludwig et al., 1988
*Paramecium*			
KCl (pH 6)	−11	1.7	Ludwig et al., 1989
LiCl (pH 6)	−24	3.4	Ludwig et al., 1989
Potassium acetate(pH 7)	+14	0.50	Ludwig et al., 1989
hVADAC1			
KCl	−11.1	1.8	Blachly-Dyson et al., 1993
hVDAC2			
KCl	−10.9	1.8	Blachly-Dyson et al., 1993
hVDAC3			
KCl	−5.4	1.3	Queralt-Martín et al., 2020

## Conductance of the Closed States of Eukaryotic Porins

The conductance of the closed state of eukaryotic porins could be evaluated from single-channel recordings extended over longer times. At voltages between 20mV and 30mV, the pores close more frequently but not too often, which means that the residual conductance associated with the closing pores could be estimated from the current recordings. This procedure allows a meaningful analysis of the conductance of the closed eukaryotic pores as it is shown in Table 3 for KCl. Included into Table 3 are also the results of this type of experiment when salts composed of different anions and cations were used. The results indicate again (similar to Figure 2) that open and closed states of the eukaryotic pores have different selectivity. This could be concluded from the observation that the single-channel conductance of the closed state of the pore was considerably smaller for Tris-HCl than for K-MES, despite a similar aqueous mobility of K^+^ and Cl^−^. This result suggested again that the closed state(s) of mitochondrial porins is cation-selective, otherwise, the relatively small conductance difference for K-MES between open and closed state and the big difference for Tris-HCl cannot be understood.

**Table 3:** Average single-channel conductance of the open and closed states of human (Porin 31HL; Benz et al., 1992) and yeast (Ludwig et al., 1988) porins in different 0.5M salt solutions.

Salt	Open state [nS]	Closed state [nS]
Human porin (Porin 31 HL)		
KCl	2.4	1.4
K-MES	0.70	0.65
Tris-HCl	1.5	0.30
Yeast porin		
KCl	2.3	1.3
K-MES	0.95	0.65
Tris-HCl	1.5	0.30

*The pH of the aqueous salt solutions was adjusted to 7.2. The protein concentration was between 5 and 10ng/ml; V_m_ =30mV, T=25°C. The single-channel conductance of the closed state was calculated by subtracting the conductance of the closing events from the conductance of the initial opening of the pores*.

## Eukaryotic Pores are Closed *In Vitro* and *in Vivo* by a Synthetic Polyanion

König et al. (1977, 1982) described effects of a 10kDa synthetic polyanion (a copolymer of methacrylate, maleate, and styrene in a 1:2:3 proportion) on mitochondrial metabolism. Dependent on its concentration, the polyanion was able to inhibit anion transport, respiration, ATPase activity, and ADP/ATP exchange activity of rat liver mitochondria (König et al., 1977, 1982). The polyanion is by far too big to enter the intermembrane space of mitochondria through the outer membrane pore and to act with inner membrane components, which means that its action on mitochondrial metabolism was something like a mystery. However, reconstitution experiments with eukaryotic porin demonstrated that the polyanion bound to porin and changed its voltage dependence (Colombini et al., 1987; Benz et al., 1988). Application of small voltages of −5mV or less negative to the cis-side of the membranes, where porin and polyanion were added, resulted already in pore closure (Benz et al., 1988; De Pinto et al., 1989a). The mitochondrial pore was always in the open configuration when positive potentials were applied to the cis-side (Benz et al., 1988).

Careful analysis of the polyanion-induced closed state of rat liver porin demonstrated that it showed an interesting analogy to the voltage-mediated closed state (see Table 4). This means that the polyanion, although it is not able to enter the pore, interacts with the gate. It pulls the gate (presumably the α-helical N-terminus) to the side of the polyanion and changes thus the voltage dependence of the gate (Benz et al., 1988). The effect of the polyanion on mitochondrial metabolism was also studied in intact mitochondria, because it allowed the evaluation of the role of the outer membrane pore on different features of mitochondrial metabolism (Benz et al., 1990; Benz and Brdiczka, 1992; Brdiczka, 1993). The addition of 30μg polyanion per mg mitochondria completely blocked adenylate and creatine kinases. Similarly, peripheral kinases, such as hexokinase and glycerolkinase, were also completely inhibited, when mitochondrial, but not external ATP, was used (Benz and Brdiczka, 1992). Disruption of the mitochondrial outer membrane by detergent completely restored the activity of all peripheral kinases, which clearly indicated that compartment formation exists in the intermembrane space of intact mitochondria (Benz and Brdiczka, 1992; Brdiczka, 1993; Gellerich et al., 1993; Ahmadzadeh et al., 1996; Brdiczka et al., 1998). These results suggest that the mitochondrial outer membrane pore could be involved in the control of mitochondrial metabolism *via* its voltage dependence (Benz and Brdiczka, 1992; Liu and Colombini, 1992). Important for this could be the close apposition of mitochondrial inner and outer membrane that a voltage across the outer membrane is induced *via* capacitive coupling of inner and outer membranes, in which also the folding of the inner membrane may be involved (Benz, 1985; Mannella et al., 2013).

**Table 4:** Average single-channel conductance of the open and the polyanion-induced closed state of rat liver porin in different 0.5M salt solutions (Benz et al., 1990).

Salt	Open state [nS]	Closed state [nS]
KCl	2.2	1.2
LiCl	1.8	0.40
K-acetate	1.1	0.85
K-MES	0.88	0.74
Tris-Cl	1.5	0.25

*The pH of the aqueous salt solutions was adjusted to 7.2. The membrane voltage was 10mV at the cis-side. The aqueous phase contained in the measurements of the closed state 0.1μg/ml polyanion added to the cis-side*.

## Renaturation and Reconstitution of Eukaryotic Porins Obtained by Heterologous Expression in Bacteria

During the first time of research into the characteristics of eukaryotic porins, these proteins were always isolated from mitochondria. However, modern research into the properties of channel-forming proteins needs very often mass production and site-directed mutagenesis of the proteins. This is possible in the case of eukaryotic porins but it is very complicated and time consuming to bring the mutated proteins back into mitochondria. Thus, it was of interest to express eukaryotic porins in bacteria and to renature eukaryotic porins for research purposes. This was possible although translation of eukaryotic porins *in vivo* and *in vitro* differs considerably. Nevertheless, two early studies describe the renaturation processes of eukaryotic porins in some detail. Pfaller et al. (1985) described for the first time the possibility to make eukaryotic porin from *N. crassa* water soluble. In this form, the protein binds to the surface of mitochondria and blocks the import of the porin precursors. The water-soluble porin may also be renatured by treatment with low doses of detergents and needs the presence of sterols in the lipid bilayer membranes (Pfaller et al., 1985). In fact, the presence of cholesterol in a ratio of five cholesterol per one polypeptide has been detected in purified eukaryotic porin using different detergents (De Pinto et al., 1989b). Sterols were also necessary when the properties of mutated *N. crassa* porin were studied in lipid bilayer membranes. Following the renaturation process of different eukaryotic porins, it seems that sterols seem to be necessary, although they may modulate the properties of the pores, in particular of plant porins (Popp et al., 1995; Carbonara et al., 1996; Mlayeh et al., 2010; Lopes-Rodrigues et al., 2020; Saidani et al., 2021). However, in mass production and functional renaturation of two human isoforms of human porin (hVDAC1 and hVDAC2) and of potato VDAC36, no indication for the need of cholesterol/sterol for porin structure and function was observed (Engelhardt et al., 2007; Lopes-Rodrigues et al., 2020; Najbauer et al., 2021). On the other hand, ergosterol interacts with eukaryotic porin of *N. crassa* (Bay and Court, 2009) and stigmasterol seems to be important for proper function of bean seed VDAC (Saidani et al., 2021) and sterols were found to be important for renaturation of VDAC from pea root plastids (Popp et al., 1995). This means presumably that it is an open question whether sterols are important for porin function and/or could only accelerate the renaturation process but are essentially not needed for the formation of some functional pores.

## Structure of the Mitochondrial Outer Membrane Pore

The presence of a voltage-dependent outer membrane pore in mitochondria was an interesting feature in research into mitochondria and attempts were made to visualize the pores. X-ray diffraction of oriented outer mitochondrial membranes from plants suggested a special location of the proteins in the membrane plane (Mannella, 1981). Further electron microscopic analysis of *Neurospora* mitochondria showed crystalline arrays of putative pores in the outer membranes if the membranes were dialyzed against low salt buffers (Mannella and Frank, 1984; Mannella, 1987). Single repeating units contained three pores, which was revealed by lipid bilayer experiments (Mannella et al., 1983). Analysis of the pores using uranyl acetate suggested that the outer membrane pore formed cylinders with an outer diameter of 5nm and an inner core of about 1.8 to 2nm (Mannella and Frank, 1984). Many attempts were made besides the electron microscopic analyses to resolve the 3D structure of the mitochondrial pore. However, all these attempts were unsuccessful for a very long time presumably because the pore was deeply buried in the outer membrane and did not show intermolecular interactions (De Pinto et al., 1987b).

Starting with eukaryotic porins from yeast (Mihara and Sato, 1985) and *N. crassa* (Kleene et al., 1987), the primary structure of many eukaryotic porins became known during the last 25years as it is discussed in part 4 of this review. Common to all of them is the length of about 280 amino acids without an obvious leader sequence and the balanced distribution of hydrophobic and hydrophilic amino acids. The genomes of many eukaryotic organisms, in particular of mammals and plants, code very often for several porins with yet not fully understood functions (Anflous and Craigen, 2004; Pinto and Messina, 2004; Graham and Craigen, 2005; Tateda et al., 2011). Secondary structure predictions suggested the existence of many β-strands within the primary sequence of eukaryotic porins similar to the situation in bacterial ones (Benz, 1994a,b). However, all mitochondrial porins known to date contain at the N-terminal end a stretch of about 20 to 25 amino acids that forms an α-helical structure, probably involved in voltage-dependent gating of the pore because its deletion leads to the loss of voltage dependence (Popp et al., 1996; Runke et al., 2006; De Pinto et al., 2007; Zachariae et al., 2012). A comparison of the many primary sequences of eukaryotic porins shows that the sequences have all a similar length, but otherwise, the homology is not very obvious because only a few amino acids are conserved (Benz, 2004). Only near amino acid 90 of most porins a triplet of the form GLK can be found, which is highly conserved but its function is unknown (Runke et al., 2000). The phylogenetic relationship of the β-barrel mitochondrial outer membrane proteins TOM40 (involved in protein transport) and eukaryotic porin was studied in detail by Young et al. (2007) and Bay et al. (2012). The authors suggested from their analysis that these proteins share a common evolutionary origin, meaning that the lineage of both protein families was co-evolutionary and formed later paralogues.

**Figure 6:**
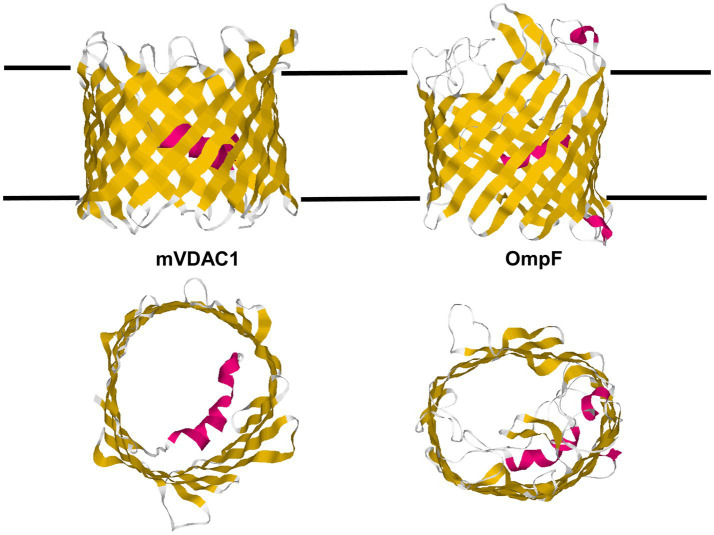
Structure of the mitochondrial outer membrane pore (mVDAC1) and an OmpF monomer of *E. coli*. β-strands within the protein structures are shown in yellow and α-helical stretches in red. The 3D structures of the proteins are shown from the side in direction to the surface of the mitochondrion and the cell (upper structures) and from the surface of the mitochondrion and the bacterial cell (structures down). mVDAC1 (PDB code: 2JK4) is the 3D structure of mouse mitochondrial porin (Ujwal et al., 2008). OmpF (PDB code: 2OMF) represents the structure of the major outer membrane protein of *E. coli* (Cowan et al., 1992).

The 3D structure of the mitochondrial pore was for a longer time a matter of debate. Based on secondary structure predictions, electrophysiology and mutagenesis several models were developed (Forte et al., 1987b; Blachly-Dyson et al., 1990, 1993; Peng et al., 1992; Benz, 1994b; Casadio et al., 2002; Anflous and Craigen, 2004). One model assumed that eukaryotic porin is exclusively formed by 16 or 18 β-strands. The other model suggested also that the pore contains β-strands, but only 12–13 strands in combination with the α-helix as part of the channel wall. At the end, three groups were successful to derive simultaneously the 3D structure of eukaryotic porins using different techniques (Bayrhuber et al., 2008; Hiller et al., 2008; Ujwal et al., 2008). Hiller et al. (2008) used the technique of solution NMR to study recombinant hVDAC1 reconstituted in detergent micelles. In this case, the location of the N-terminus was not resolved. Bayrhuber et al. (2008) derived the 3D structure of hVDAC1 from a combination of NMR-spectroscopy and X-ray crystallography. Ujwal et al. (2008) succeeded to crystallize murine VDAC1 (mVDAC1). The three studies agreed in the basic structure of the mitochondrial pore that forms a β-barrel with 19 β-strands. Two structures show the location of the N-terminal α-helix horizontally midway in the pore, restricting its size (Bayrhuber et al., 2008; Ujwal et al., 2008). This means that the α-helix has a strategic position to control the passage of metabolites and ions through the mitochondrial pore. This structure was criticized and Colombini (2012) insisted in the structure of VDAC with one α-helix and 13 β-strands tilted at a 46° angle toward the surface of the mitochondrial outer membrane. However, the structure of VDAC shown in Figure 6 has many times been realized that we can consider it as the real structure of the eukaryotic pore.

Figure 6 shows the schematic structure of mouse VDAC as it was obtained by X-ray crystallography (Ujwal et al., 2008) in comparison with the 3D structure of the most abandoned bacterial porin OmpF of *E. coli* (Cowan et al., 1992). The mitochondrial pore is formed by 19 β-strands (18 are antiparallel and β-strands one and 19 are parallel) in contrast to 16 antiparallel β-strands of OmpF. It is clear from a comparison of the two 3D structures that the architecture of the two outer membrane pores is quite similar. This has presumably to do with the history of bacterial and mitochondrial outer membrane pores. It has presumably also to do with translation and assembly of both pores. The β-strands of both β-barrel cylinders are tilted by 30–40° toward the surface of the membranes. The dimensions of the eukaryotic porin are 35 Ă for the height and 40 Ă for the width. The N-terminal α-helix (amino acids 1–21) is located inside the β-barrel cylinder and acts there as a gate, but also as a stabilizing element for the mitochondrial pore similar to the external loop 3 of OmpF that is folded inside the bacterial pore (Cowan et al., 1992; Ujwal et al., 2008). Despite the location of the N-terminus inside the eukaryotic pore, it has a high ion permeability. It is approximately the same as OmpF trimers (Benz, 1994a,b). It is noteworthy, that Tom40, the major component of the mitochondrial outer membrane import machinery is also a member of the VDAC-family and shows the same structure of 18 antiparallel β-strands and one pair of parallel β-strands (Zeth and Zachariae, 2018). The most interesting point of the comparison of bacterial and mitochondrial porins is the fact that bacterial outer membrane pores have only passive properties, whereas mitochondrial porins adopted during evolution an active role in mitochondrial metabolism.

## Author Contributions

The author confirms being the sole contributor of this work and has approved it for publication.

## Conflict of Interest

The author declares that the research was conducted in the absence of any commercial or financial relationships that could be construed as a potential conflict of interest.

## Publisher’s Note

All claims expressed in this article are solely those of the authors and do not necessarily represent those of their affiliated organizations, or those of the publisher, the editors and the reviewers. Any product that may be evaluated in this article, or claim that may be made by its manufacturer, is not guaranteed or endorsed by the publisher.
